# Shotgun Metagenomic Analysis of Gut Microbiota and Antibiotic Resistance Genes in a High-Fat Diet Mouse Model Treated with Heat-Killed *Lactiplantibacillus plantarum* beLP1

**DOI:** 10.3390/microorganisms14050944

**Published:** 2026-04-22

**Authors:** Ranjith Kumar Manoharan, Hyun-Dong Shin, Yura Lee, Sunhwa Baek, Eunjung Moon, Youn Bum Park, Junhui Cho, Im-Joung La, Dong Ha Lee, Kwon-Il Han, Sathiyaraj Srinivasan

**Affiliations:** 1 Research & Development Center, Bereum Co., Ltd., Wonju 26361, Republic of Korea; 2 Atomy R&D Center, Gongju 32511, Republic of Korea; 3 Dx&Vx Co., Ltd., Geumchoen-gu, Seoul 08513, Republic of Korea; 4 Department of Bio & Environmental Technology, College of Natural Science, Seoul Women’s University, 623 Hwarangno, Nowon-gu, Seoul 01797, Republic of Korea

**Keywords:** *Lactiplantibacillus plantarum* beLP1, postbiotics, high-fat diet, SCFA, metagenomics

## Abstract

The gut microbiota is a central regulator of metabolic function, and its disruption by a high-fat diet (HFD) is strongly linked to obesity and metabolic impairment. This study evaluated the potential of heat-killed *Lactiplantibacillus plantarum* beLP1 (beLP1^®^) in alleviating HFD-induced metabolic and microbial imbalances in mice. Male C57BL/6N mice were fed an HFD for 10 weeks, with or without daily oral supplementation of beLP1 (≥3 × 10^10^ cells). Compared with untreated HFD mice, beLP1 supplementation reduced serum triglycerides by 35% and lowered liver enzymes AST and ALT by 17% and 36%, respectively. Blood glucose levels remained similar to the HFD group throughout the study period. Shotgun metagenomic analysis revealed that beLP1 restored gut microbial diversity, increased beneficial taxa such as *Akkermansia* and *Faecalibaculum* high. and reduced pro-inflammatory species including *Streptococcus* sp., *Mucispirillum schaedleri* and *Clostridium cocleatum*. These microbial changes were associated with partial normalization of the Firmicutes/Bacteroidota ratio and improvements in antibiotic resistance gene (ARG) profiles. Specifically, in silico analysis of the short-chain fatty acid (SCFA) synthesis pathways indicated that the potential for acetate and propionate production was maximized in the beLP1 group, resulting in the highest relative abundance among all groups. This functional enhancement directly correlated with the enrichment of key SCFA-producing taxa, particularly *Akkermansia muciniphila,* confirming that increased bacterial abundance suggests an enhanced functional potential for SCFA production. Furthermore, beLP1^®^ induced a selective modulation of gut ARGs, significantly reducing specific subtypes such as tetracycline and multidrug efflux genes, despite a slight increase in vancomycin resistance markers. Overall, our findings suggest that *beLP1^®^* attenuated the rate of body weight gain during the initial weeks of HFD exposure and significantly improved markers of hepatic stress and lipid metabolism.

## 1. Introduction

The gut microbiota functions as a key regulator of host metabolic homeostasis, influencing energy harvest, immune signaling, intestinal integrity, and neuroendocrine crosstalk through the gut–brain axis [1,2,3]. Dietary patterns, particularly chronic consumption of high-fat diets (HFD), are known to disrupt the structure and function of this ecosystem profoundly. HFD exposure frequently causes microbial disequilibrium, demonstrated by decreased community diversity, altered taxonomic structure, and impaired intestinal barrier function, which are linked to obesity, insulin resistance, and non-alcoholic fatty liver disease (NAFLD) [4,5]. A frequently reported microbiome shift under HFD is an increased Firmicutes/Bacteroidota ratio [6]. Germ-free and fecal microbiota transplantation (FMT) studies have confirmed that gut microbial alterations can transmit obesity and insulin resistance phenotypes, underscoring the causal role of the microbiota in metabolic disease pathogenesis [7]. Probiotic interventions have shown promise in restoring microbial balance, with *Lactiplantibacillus plantarum* (formerly *Lactobacillus plantarum*) emerging as one of the most well-characterized species for its immunomodulatory, anti-obesity, and gut barrier-enhancing properties [8,9]. However, challenges related to the viability, stability, and host-specific effects of live probiotics have shifted attention toward postbiotics, which are non-viable microbial cells or their components that retain functional activity [10]. The concept of postbiotics encompasses both purified microbial metabolites (e.g., SCFAs, bacteriocins, and cell wall fragments) and inactivated whole-cell preparations, particularly those generated through heat-killing [11,12].

SCFAs such as acetate, propionate, and butyrate have emerged as key microbial metabolites that modulate host metabolic signaling and immune responses [13]. These microbial products are primarily produced by gut bacteria fermenting indigestible carbohydrates, exerting beneficial effects on energy homeostasis, gut barrier integrity, and inflammatory responses [14]. Given their significant impact, understanding the specific pathways involved in SCFA production and how dietary interventions or postbiotic treatments modulate them is crucial for developing effective therapeutic strategies [15]. While metabolite-based postbiotics can offer targeted functional benefits, such as butyrate-mediated energy regulation or anti-inflammatory cytokine induction, intact whole-cell heat-killed preparations deliver a broader spectrum of activity. These effects are partially mediated by bacterial structural components such as peptidoglycans, lipoteichoic acids, and surface-associated proteins, which interact with host pattern-recognition receptors (PRRs) to maintain epithelial homeostasis and immunological control [16,17]. Importantly, the health effects of postbiotics are highly strain-specific and may not be replicated by isolated metabolites alone [18].

Emerging evidence has demonstrated the potential of heat-killed *L. plantarum* strains to attenuate diet-induced obesity, modulate host lipid metabolism, suppress pro-inflammatory cytokines, and enhance gut microbiota resilience [12,19]. Despite this, many promising strains remain underexplored. One such candidate is *L. plantarum* beLP1 (non-viable), a heat-killed strain whose physiological and microbial effects in HFD-induced obesity have not yet been characterized. The use of whole-cell postbiotics like beLP1 offers the advantage of combining metabolic, immunological, and ecological effects into a single intervention [12]. Additionally, there is growing concern that HFD-induced dysbiosis may contribute to the expansion of antibiotic resistance genes (ARGs) within the gut microbiome. Notably, genes encoding efflux pumps, such as *bcrA*, *cdeA*, and *msbA*, are often enriched in dysbiotic conditions and are associated with reduced microbiome stability and resilience [20,21]. Therefore, interventions that not only restore microbiota balance but also prevent ARG proliferation represent a particularly valuable therapeutic strategy.

In this study, we examined the effects of oral administration of heat-killed *L. plantarum* beLP1^®^ in a mouse model of HFD-induced obesity. We evaluated its impact on body weight, lipid and glucose metabolism, liver function, gut microbial composition and diversity, and ARG abundance. By characterizing the whole-cell postbiotic activity of beLP1^®^, this study provides a comprehensive analysis of its strain-specific metabolic benefits and highlights its potential as a safe, multifunctional candidate for microbiome-targeted management. Specifically, we investigated the impact of beLP1^®^ on short-chain fatty acid (SCFA) pathways, focusing on the microbial enzymatic machinery responsible for acetate biosynthesis in a high-fat diet model through shotgun metagenomic analysis and bioinformatics tools, including *eggNOG* and *KEGG COG*. Specifically, this research aimed to elucidate how beLP1 influences the microbial enzymatic machinery responsible for acetate biosynthesis, thereby offering mechanistic insights into its potential metabolic benefits. The investigation employed shotgun metagenomic analysis to comprehensively identify and quantify genes associated with SCFA production, especially those involved in acetate synthesis, across different dietary and treatment groups.

## 2. Materials and Methods

### 2.1. Animal Model and Experimental Structure

The detailed experimental design and timeline are illustrated in Figure S1. Seven-week-old male C57BL/6N mice (Orient Bio Inc., Seongnam, Republic of Korea) were housed under controlled environmental conditions (22 ± 2 °C; 55 ± 15% humidity; 12-h light/dark cycle) with free food and drink [22]. Animals were acclimatized for three weeks before dietary intervention as described earlier [22]. Following acclimation, mice were randomized into three groups: normal diet (ND, *n* = 5 + 3), high-fat diet (HFD, *n* = 10 + 3) 60% kcal fat diet (D12492, Research Diets, New Brunswick, NJ, USA), and HFD supplemented with beLP1^®^ (*n* = 10 + 3; ≥3 × 10^10^ cells/day by oral gavage) (Figure S1). HFD feeding continued until mice reached ≥30 g of body weight. Following three weeks of HFD intake, baseline blood and fecal samples were obtained and three animals per group were excluded from subsequent assessments to reduce stress caused by repetitive handling. All procedures were approved by the Woojung Bio, Inc. (Hwaseong, Republic of Korea) IACUC (IACIC2403-005, 17 July 2024) and were conducted at Hu-mic, Inc. (Jacksonville, FL, USA) in compliance with national welfare regulations. The remaining 10 mice in each group were used in all major analyses, and no animals were removed after the trial began. The heat-killed *L. plantarum* beLP1 (postbiotic) used in this study was a standardized commercial preparation provided by Bereum Co., Ltd. (Wonju, Republic of Korea), as utilized in previous studies [23,24]. The product holds GRAS and FDA NDI certifications, and complete inactivation of the bacteria was confirmed prior to administration via the manufacturer’s standard quality control plating assays.

### 2.2. Physiological Monitoring and Tissue Collection

Body weight was assessed twice a week, and weekly food intake was calculated to ascertain cumulative consumption. At week 10, animals were euthanized via chloral hydrate anesthesia (400 mg/kg BW). Liver and adipose depots (subcutaneous, gonadal, perirenal, mesenteric, and pericardial) were dissected, weighed, and snap-frozen at −80 °C for further analysis.

### 2.3. Biochemical Assays

Blood samples were collected at four different time points: pre-induction, pre-diet, week 6, and week 10. Serum was separated (3000 rpm, 20 min) and stored at −80 °C. Serum ALT, AST, and triglyceride concentrations were quantified using an automated biochemical analyzer (Hitachi 7600-210, Tokyo, Japan). Fasting blood glucose was assessed in all remaining animals at identical time points [22].

### 2.4. Fecal Sampling and DNA Preparation

Fecal pellets were collected at baseline, week 6, and week 10 and then stored at −80 °C. DNA was extracted using the QIAamp PowerFecal DNA Kit (Qiagen, Hilden, Germany). Concentration was quantified with a Qubit™ dsDNA HS Assay Kit (Thermo Fisher Scientific, Waltham, MA, USA) on a Qubit 3 fluorometer (Thermo Fisher Scientific, Waltham, MA, USA), purity was assessed by NanoDrop spectrophotometry (260/280 ratio 1.8–2.0), and integrity was evaluated on an Agilent 4200 TapeStation. Genomic DNA was sheared to approximately 200 bp using a Bioruptor (Diagenode, Seraing, Belgium), purified with a QIAquick PCR Clean-Up Kit (Qiagen, Hilden, Germany), and re-quantified.

### 2.5. Shotgun Metagenomic Sequencing and Analysis

Genomic DNA from ND, HFD, and HFD + beLP1^®^ groups, each *n* = 5 samples, was chosen for shotgun metagenomic sequencing (NovaSeq 6000, Illumina, San Diego, CA, USA; paired-end, 150 bp) [22]. Sequencing base calling was performed using Illumina’s Real-Time Analysis (RTA) software (RTA version 1.18.66.3; Illumina, Inc., San Diego, CA, USA), and FASTQ files were generated with either bcl2fastq2 or bclconvert. Taxonomic classification used a multi-tool pipeline: Kraken2 for initial assignment, Bracken for abundance refinement, Centrifuge for additional genome-based classification, and MetaPhlAn for marker gene-based profiling.

### 2.6. Identification of ARGs and MGEs

Antimicrobial resistome profiling involved aligning unigenes to the CARD database (v2.0.1) using BLAST package (version 2.9.0+) with an e-value cutoff of ≤1 × 10^−30^. The relative abundance of microbial functional genes was calculated using the TPM (Transcripts Per Million) method to allow for accurate cross-sample comparisons, and resistance gene characteristics were interpreted using the Resistance Gene Identifier (RGI).

### 2.7. Functional Potential and SCFA Pathway Analysis

The functional potential of the gut microbiome was determined by analyzing shotgun metagenomic reads using the DIAMOND/MEGAN5 pipeline. All paired and unpaired reads were annotated against the EggNOG database (utilizing the October 2016 protein accession mapping file) and the NCBI-NR protein database (using the June 2018 GI to NCBI taxonomy mapping files). The generated functional catalog was used to classify genes into comprehensive categories, including the Clusters of Orthologous Groups (COGs). For targeted metabolic assessment, the relative potential for SCFA synthesis pathways (acetate, propionate, and butyrate oxidation, and methanogenesis) was quantified. This was achieved by compiling a specific functional catalog based on the relative abundance of key enzymes and genes identified within the Kyoto Encyclopedia of Genes and Genomes (KEGG) database for the pathways of interest. This approach allowed for the assessment of functional potential per genus by combining taxonomic and functional data.

### 2.8. Statistical Analysis

Normality was assessed before analysis. Statistical analyses used one-way ANOVA with Dunnett’s correction for normally distributed data, and Kruskal–Wallis tests with Dunn’s post hoc comparisons for non-parametric variables. Statistical significance was set at *p* < 0.05, and all analyses were performed using R (version 4.3.3).

## 3. Results

### 3.1. Body Weight in HFD-Induced Obese Mouse

**Figure 1:**
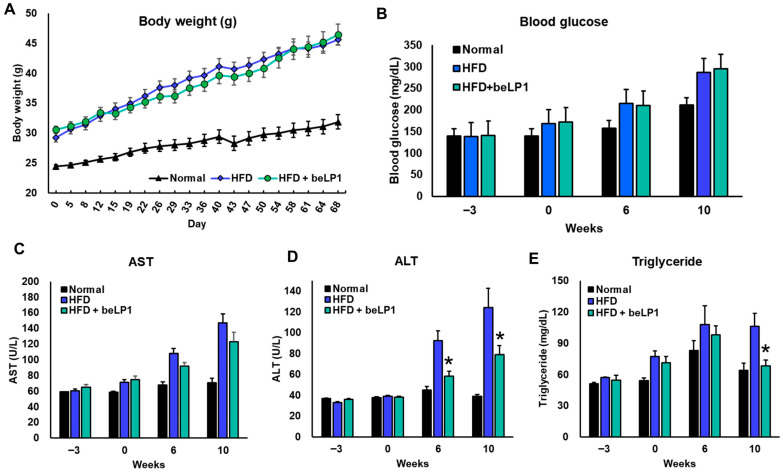
Effects of beLP1 on metabolic indices in HFD-fed mice. Body weight (**A**), blood glucose levels (**B**), and liver function biomarkers such as AST (**C**), ALT (**D**) and Triglyceride (**E**) were assessed at baseline, week 6, and week 10 across ND, HFD, and HFD + beLP1 groups (*n* = 5 for ND; *n* = 10 for HFD and HFD + beLP1). Data are presented as mean ± SEM. Statistical significance was determined using a one-way ANOVA followed by Tukey’s multiple comparison test using GraphPad Prism 8.4.3 (* *p* < 0.05 compared to the HFD group).

Figure 1A illustrates body weight progression over the 10-week study period. At baseline (Day 0), the ND group weighed approximately 24 g, while the HFD-acclimated groups started at approximately 29–30 g. The significant difference in body weight gain was observed during the initial weeks (e.g., Weeks 1–4), but the final body weights in Week 10 were comparable. The mice in the HFD group gained significantly more weight (~42 g), whereas those receiving beLP1 had a noticeably lower weight (~39 g), indicating a 7.14% reduction in weight gain by day 40 (Figure S2). However, this difference between the HFD and the HFD+ beLP1^®^ group was gradually reduced after 6 weeks. By week 10, the HFD group reached an average of 45.98 ± 0.8 g, while the beLP1^®^-treated group recorded a similar value of 45.74 ± 1.0 g, indicating no change in weight gain. The ND group remained significantly lower at 31.02 ± 0.6 g. While beLP1^®^ did not completely prevent HFD-induced weight gain, it slowed the rate of increase compared to untreated HFD mice, suggesting a partial protective effect. As shown in Figure 1B, fasting blood glucose levels increased steadily in the HFD group, reaching 286.4 ± 36.8 mg/dL by week 10, a 35% rise from baseline. The beLP1^®^ group demonstrated a similar trend to the HFD group, peaking at 294.9 ± 24.3 mg/dL, indicating no significant attenuation of fasting blood glucose levels. While beLP1^®^ did not significantly alter static markers of glucose homeostasis, such as fasting blood glucose, it exhibited significant efficacy in lowering lipid-related metabolic markers. Liver enzyme analysis (Figure 1C,D) demonstrated that by week 10, HFD feeding substantially elevated AST and ALT levels to 147.17 ± 36.11 U/L and 124.07 ± 57.86 U/L, respectively, compared to the ND group (AST: 70.76 ± 12.21 U/L; ALT: 38.98 ± 3.91 U/L). When comparing these same time points, supplementation with beLP1^®^ attenuated this HFD-induced elevation, resulting in week 10 AST levels of 122.95 ± 37.42 U/L and ALT levels of 78.83 ± 27.23 U/L. While liver enzymes in the beLP1^®^ group showed an initial dip at week 6 before rising slightly by week 10, the final week 10 levels remained lower than those of the untreated HFD group, indicating a sustained, partial protective effect on hepatic function. Triglyceride levels (Figure 1E) also rose in HFD-fed mice (106.38 ± 6.7 mg/dL) but were significantly lower in the beLP1 group at 68.55 ± 5.8 mg/dL, an improvement of approximately 35.5% compared to the HFD group.

Collectively, these data support the conclusion that beLP1 supplementation improves multiple metabolic parameters, including hepatic enzyme levels and triglyceride accumulation in mice subjected to an HFD.

### 3.2. Effect of beLP1 on Organ and Adipose Tissue Weight in HFD-Induced Obese Mice

**Figure 2:**
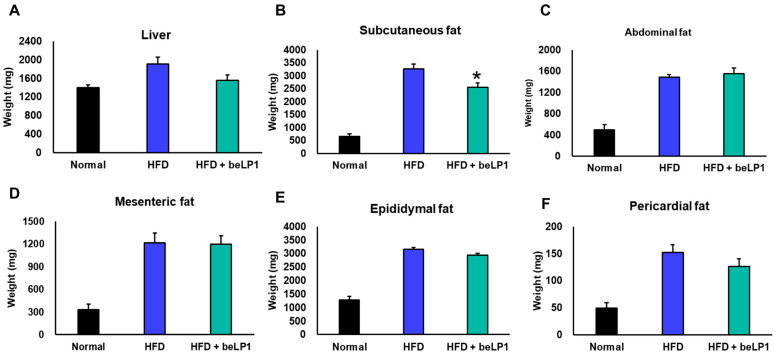
Effects of beLP1 on organ and adipose tissue weights in HFD-fed mice. The weights of the liver (**A**) and various fat depots (subcutaneous (**B**), abdominal (**C**), mesenteric (**D**), epididymal (**E**), and pericardial (**F**)) were measured after 10 weeks of beLP1 supplementation (*n* = 5 for ND; *n* = 10 for HFD and HFD + beLP1). Data are presented as mean ± SEM. Statistical significance was determined using a one-way ANOVA followed by Tukey’s multiple comparison test (* *p* < 0.05 vs. HFD group).

To evaluate the effects of beLP1^®^ on organ and fat tissue accumulation under HFD conditions, the weights of the liver and five major fat depots were measured at the end of the 10-week intervention period (Figure 2A–F). HFD feeding significantly increased the weights of all analyzed tissues compared to the ND group, reflecting classic features of diet-induced obesity. However, beLP1^®^ supplementation demonstrated a trend toward reducing specific visceral fat depots, notably subcutaneous and pericardial fat, though not all depots reached statistical significance.

Liver weight increased substantially in the HFD group compared to the ND group. Notably, beLP1^®^-treated mice exhibited an 18.5% reduction in liver weight relative to HFD controls and were 10% heavier than ND-fed mice (Figure 2A), suggesting that beLP1 protects against hepatic lipid overload and steatosis.

Subcutaneous fat mass (Figure 2B) was markedly higher in HFD-fed mice but was significantly reduced by 21.7% following beLP1^®^ administration. Abdominal fat (Figure 2C) also showed an increase of 4.8% in the beLP1^®^ group compared to HFD; however, this difference was not statistically significant.

Mesenteric fat, a depot linked to intestinal inflammation and metabolic complications, was reduced by 1.3% with beLP1^®^ treatment (Figure 2D). Similarly, epididymal fat mass (Figure 2E), a key visceral depot, was 6.8% lower in the beLP1 group, indicating mitigation of central obesity.

Pericardial fat, often associated with cardiovascular risk, was reduced by 17.02% in beLP1^®^-treated mice (Figure 2F), suggesting a potential protective effect against obesity-related cardiac complications. Overall, these data demonstrate that beLP1^®^ administration potentially attenuates HFD-induced fat accumulation and subsequent organ weight gain. These effects are consistent with previously reported anti-obesity properties of postbiotics, including heat-killed strains, and support the role of beLP1^®^ as a functional intervention to mitigate adipose tissue hypertrophy and hepatic steatosis in the context of dietary obesity.

### 3.3. Metagenomic Diversity and Composition Shifts Induced by beLP1

**Figure 3:**
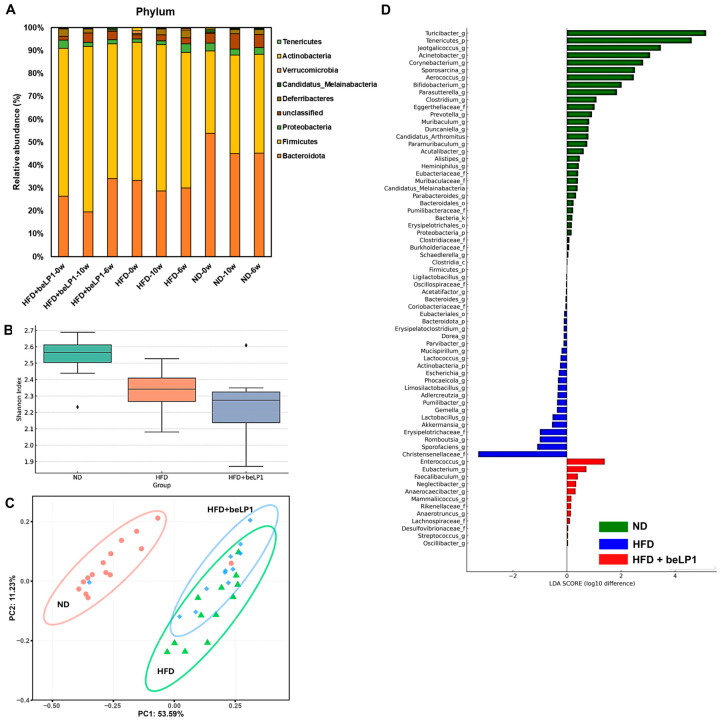
Gut microbiome shifts after 10 weeks of beLP1 supplementation: (**A**) phylum-level taxonomic profiles; (**B**) Shannon diversity indices; (**C**) PCoA based on Bray–Curtis distances; (**D**) LEfSe analysis using a log10 LDA threshold. Ecological analyses of the metagenomic data were primarily conducted using R (v4.3.3). Alpha diversity (Shannon index) was calculated to assess community richness and evenness within samples. For beta diversity (**C**), Principal Coordinates Analysis (PCoA) was performed based on Bray–Curtis dissimilarity matrices using the vegan R package to evaluate structural variations in microbial communities across groups; statistical separation was tested using PERMANOVA (adonis function, permutations = 999). Circle dots denotes ND, whereas triangle and diamond symbol denotes HFD and HFD + beLP1. To identify specific microbial biomarkers driving group differences (**D**), Linear discriminant analysis Effect Size (LEfSe) was employed, with a stringent logarithmic LDA score threshold set at >3.0 and an alpha value of 0.05 for the factorial Kruskal–Wallis test.

To assess the impact of beLP1^®^ on gut microbiome structure, we analyzed microbial composition and diversity at multiple levels. HFD feeding significantly altered microbial community structure, which was partially restored by beLP1^®^ supplementation (Figure 3).

At the phylum level (Figure 3A), HFD-fed mice exhibited a typical dysbiotic profile, characterized by a reduction in Bacteroidota and an increase in Firmicutes. Notably, beLP1^®^ administration partially reversed this trend, leading to a transient increase in Bacteroidota and a modest decrease in Firmicutes, particularly evident at week 6. The Shannon index-based alpha diversity analysis (Figure 3B) demonstrated a decrease in microbial diversity in HFD-fed mice compared to ND controls. Boxplot analysis (Figure S3) further demonstrated reduced inter-sample distances in the beLP1 group compared to the HFD group, suggesting greater microbial stability and reduced variability across individuals. Beta-diversity, assessed using Principal Coordinates Analysis (PCoA) based on Bray–Curtis dissimilarity (Figure 3C), shows a minor intermediate trend, remaining largely overlapping with the HFD group, suggesting only partial compositional shifts rather than full restoration. This intermediate positioning suggests that beLP1^®^ can modulate microbiome structure toward a healthier state, though not completely reverting to the ND profile.

LDA scores for the differentially abundant bacterial taxa between three groups (log_10_ difference) (Figure 3D) revealed significant differences in gut microbial composition among the ND, HFD, and HFD+ beLP1^®^ groups. Notably, the ND group was enriched with genera such as *Turicibacter*, *Tennericutes*, and *Corynebacterium*, while *Christensenellaceae* and *Akkermansia* were predominant in the HFD group, and *Enterococcus* and *Faecalibaculum* were dominant in the HFD+ beLP1^®^ group compared to other groups.

While HFD feeding induced typical dysbiotic shifts, including an altered Firmicutes/Bacteroidota ratio, beLP1^®^ supplementation did not induce a broad-spectrum ecological restoration of these baseline community metrics. In fact, alpha diversity (Shannon index) was modestly lower in the beLP1^®^-treated group (Figure 3B). Therefore, these compositional changes reflect a targeted restructuring of the microbiome specifically enriching highly functional, SCFA-producing species to establish a protective metabolic environment rather than a complete return to the pre-induction taxonomic profile.

### 3.4. Genus-Level and Species-Level Modulation by beLP1 in HFD-Induced Obese Mice

**Figure 4:**
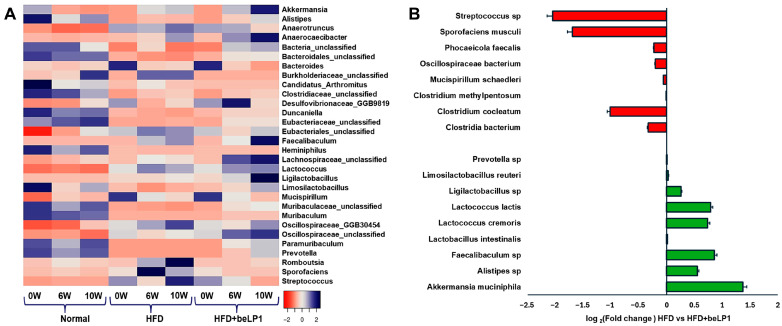
Microbial composition across fecal samples. (**A**) Genus-level heatmap representing the relative abundance of the top 30 taxa at different time points across ND, HFD, and HFD + beLP1 groups. (**B**) Log2 fold-change comparison of specific bacterial taxa between the HFD and HFD + beLP1 groups after 10 weeks of supplementation. Error bars represent standard deviation (SD). Statistical significance was determined using one-way ANOVA with Dunnett’s correction for normally distributed data, and Kruskal–Wallis tests with Dunn’s post hoc comparisons for non-parametric variables, conducted in R (v4.3.3).

Genus-level and species-level profiling of gut microbiota in response to beLP1^®^ supplementation revealed significant shifts in microbial composition, as shown in Figure 4. Genus-level comparisons indicated increased abundance of several taxa including *Akkermansia*, *Alistipes*, *Faecalibaculum*, *Lactococcus*, *Ligilactobacillus*, *Limosilactobacillus*, and members of *Lachnospiraceae* in the beLP1-treated group relative to HFD controls (Figure 4A). Fold change analysis (Figure 4B) demonstrated that beLP1^®^ supplementation selectively enriched beneficial bacterial genera while suppressing inflammation-associated and dysbiotic taxa. Beneficial species enriched in the beLP1^®^ group included *Akkermansia muciniphila*, *Alistipes* sp., *Faecalibaculum sp.*, *Lactococcus lactis*, *Lactococcus cremoris*, *Lachnospiraceae bacterium*, *Ligilactobacillus murinus*, and *Limsolactobacillus reuteri*. Among these, *A. muciniphila* showed the highest positive shift, with a log_2_ fold change of +1.3, indicating nearly a 2.5-fold enrichment over the HFD group. Similarly, *Alistipes* sp showed positive shift, with a log_2_ fold change of +0.6 (Figure 4B and Figure S4). Likewise, *Faecalibaculum* sp. and *Lactococcus* spp. (lactis and cremoris) were significantly enriched (log_2_ FC > +0.6). *Lachnospiraceae bacterium*, *Prevotella sp.*, and *Oscillibacter sp.* also showed mild but consistent increases in the beLP1 group (Figure 4A and Figure S4). Many of these taxa support beLP1^®^’s role in restoring gut microbial homeostasis under obesogenic conditions.

Conversely, several inflammation-associated taxa were suppressed by beLP1. Notably, *Streptococcus* (−2 fold), *Sporofaciens musculi* (−1.7 fold), and *Erysipelatoclostridium cocleatum* (formerly *Clostridium cocleatum*; −1 fold) showed substantial decreases, highlighting the anti-inflammatory properties of the intervention. These bacteria are often associated with gut dysbiosis and may contribute to metabolic disorders, such as obesity and insulin resistance. The reduction in these taxa supports the hypothesis that beLP1^®^ helps restore a more balanced gut microbiota in HFD-fed mice.

Additionally, *Lactobacillus* species, including *L. lactis* and *L. cremoris*, showed positive shifts (>+0.7), reinforcing the role of beLP1 in promoting gut microbiota known for their probiotic potential. These species-specific shifts suggest that beLP1^®^ selectively promotes the expansion of beneficial, SCFA-producing bacteria while suppressing harmful or opportunistic taxa associated with HFD-induced dysbiosis (Figure S4). This microbial remodeling may underpin the observed metabolic benefits of beLP1^®^, including improved glycemic control, reduced adiposity, and lower liver enzyme levels.

### 3.5. Functional Potential of Gut Microbiota: SCFA Pathway Analysis (EggNOG Database)

This section presents the quantitative assessment of the gut microbial community’s predicted metabolic capacity for SCFA synthesis pathways, based on functional annotation derived from shotgun metagenomic sequencing data analyzed using the EggNOG database.

#### 3.5.1. Relative Composition of Predicted SCFA Synthesis Pathways

**Figure 5:**
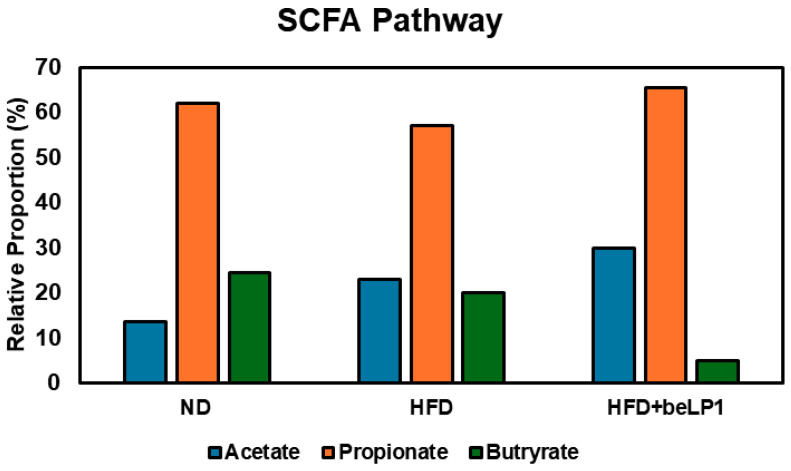
Relative composition of SCFA pathways across experimental groups. The stacked bar plots show the proportional contributions of the acetate, propionate, and butyrate pathways in the colon contents of mice fed a normal diet (ND), a high-fat diet (HFD), or an HFD supplemented with beLP1, based on in silico analysis. Numerical labels represent the relative percentage contribution of each SCFA synthesis pathway to the total functional potential. Pathway potential was quantified using TPM-normalized gene abundances.

Functional analysis of the metagenome revealed distinct, group-specific profiles in the relative proportion of genes annotated for acetate, propionate, and butyrate synthesis pathways (Figure 5). In the ND group, the predicted metabolic landscape reflected a profile typical of healthy colonic energy balance, dominated by propionate synthesis potential, accounting for approximately 62.0% of the total SCFA pathway genes, followed by butyrate at approximately 24.5%. Acetate synthesis contributed the lowest relative proportion, estimated at 13.5% (Figure 5).

HFD supplementation resulted in a significant functional shift away from the healthy profile. The relative abundance of the acetate synthesis pathway genes increased substantially in the HFD group, rising to approximately 23.0% (Figure 5). This HFD-driven increase in acetate potential coincided with corresponding reductions in both propionate and butyrate potentials, which fell to approximately 57.0% and 20.0%, respectively (Figure 5). This outcome suggests an HFD-induced functional dysbiosis favoring specific primary fermenters and potentially compromising the secondary fermentation cascade.

Oral administration of heat-killed beLP1 (HFD+ beLP1^®^ group) did not reverse the HFD-induced shift toward the ND profile but instead amplified the acetate production potential. The relative abundance of the acetate synthesis pathway potential reached its highest point across all groups. This enrichment represents a robust absolute increase of approximately 9.0% relative to the untreated HFD group. Similarly, there was a slight increase in propionate compared to HFD and ND groups (Figure 5). This maximal functional shift confirms a highly targeted impact of the postbiotic intervention on metabolic functionality. Conversely, the beLP1^®^ intervention led to the lowest observed relative proportions for butyrate (15.0%) synthesis pathways across all experimental groups (Figure 5). This pattern indicates a functional remodeling centered predominantly on maximizing acetate and propionate generation capability within the HFD-conditioned environment.

#### 3.5.2. Predicted Functional Profile (COG Analysis)

Functional annotation of the metagenome using the EggNOG database also provided insights into the functional potential of the gut microbial community by classifying genes into Clusters of Orthologous Groups (COG). The relative abundance distribution across the 23 main COG functional categories (Figure S5) showed clear metabolic shifts induced by the HFD and subsequent modulation by beLP1^®^.

Hierarchical clustering of the functional profiles grouped the Normal Diet (ND) samples separately from the HFD and beLP1^®^-treated (HFD + beLP1^®^) samples, confirming that the HFD regimen induced a profound and persistent change in the overall functional potential of the microbiome. The HFD groups (HFD-W6 and HFD-W10) exhibited a distinct functional profile compared to the ND groups, characterized by visible alterations in key metabolic categories. Specifically, Energy production and conversion (COG), which dominates the ND profile, appeared comparatively lower in the HFD groups.

The beLP1^®^ intervention led to specific functional modulation within the HFD context. Functional profiling via COG categories (Figure S5) revealed distinct metabolic shifts driven by dietary intervention. Specifically, pathways related to Energy production and conversion exhibited lower relative abundance in the ND profile compared to the HFD-fed groups, where these pathways appeared notably enriched. Furthermore, the most prominent diet-induced alterations were observed in pathways such as Nucleotide transport and metabolism, which demonstrated distinct shifts between the ND and HFD groups. Regarding temporal stability, the hierarchical clustering analysis indicated that the untreated HFD-W6 and HFD-W10 samples clustered more closely together than the HFD+ beLP1^®^-W6 and HFD+ beLP1^®^-W10 groups (Figure S5). This clustering pattern suggests that while the HFD-induced microbiome remains functionally static over time, beLP1^®^ supplementation introduces ongoing, progressive functional shifts across the 10-week intervention period. The changes in the COG profile visually confirm that the beLP1^®^ intervention selectively influences the metabolic capacity of the gut microbiome, providing context for the targeted changes observed in the SCFA pathways.

### 3.6. Antibiotic Resistance Genes (ARGs) Analysis

#### ARG Distribution and Co-Occurrence

**Figure 6:**
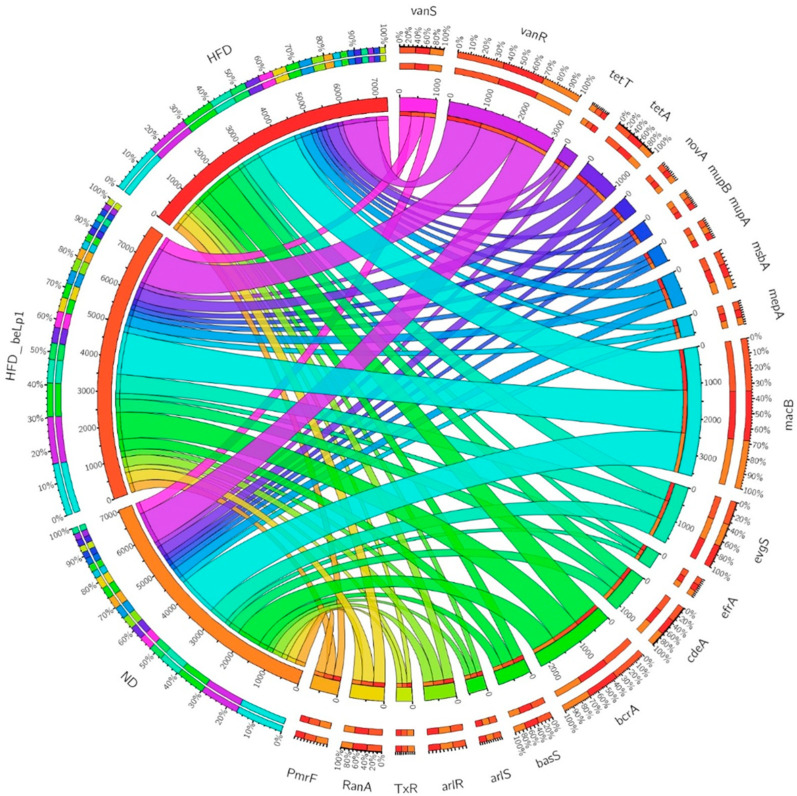
Circular chord diagram showing the distribution and co-occurrence of antibiotic resistance genes (ARGs) among experimental groups. Each arc on the outer circle represents an ARG or treatment group (ND, HFD, HFD + beLP1). The width of the inner connecting ribbons illustrates the proportional contribution of a specific group to the total detected abundance of that resistance gene.

Shotgun metagenomic sequencing revealed distinct shifts in the gut resistome in response to dietary interventions. The circular chord diagram (Figure 6) illustrates the abundance and co-occurrence patterns of ARGs among the ND, HFD, and HFD+ beLP1^®^ groups. Mice in the HFD group exhibited a markedly higher ARG load than those in the ND group. Metagenomic profiling of the resistome (Figure 6) revealed distinct distributional shifts in specific ARGs across the dietary groups, including those associated with multidrug efflux pumps and tetracycline resistance, reflecting the broader taxonomic restructuring driven by the HFD and subsequent beLP1^®^ intervention. These genes formed dense linkages with the HFD sector in the chord diagram, underscoring their dominant role in the resistome under obesogenic dietary conditions.

The administration of beLP1^®^ induced a selective and bidirectional modulation of the gut resistome, characterized by gene-specific shifts across various resistance mechanisms rather than a uniform reduction (Table 1). A comparative analysis between the HFD and HFD+ beLP1^®^ groups revealed that within the antibiotic efflux category, which exhibited the most heterogeneous response, the multidrug efflux gene *msbA* was slightly reduced by 1.6% (355.4 to 349.6), whereas pronounced increases were observed in the multidrug resistance gene *bcrA* (866.4 to 873.0) and other transporters, including the macrolide-like ABC transporter *oleC* (147.8 to 173.0) and the macrolide pump macB (1309.2 to 1326.8). Furthermore, while beLP1^®^ treatment did not further exacerbate the HFD-induced levels of the efflux-associated genes *golS* (126.6) and *cdeA* (426.4), these markers remained markedly elevated relative to the ND baseline (84.0 and 368.8, respectively), indicating a failure to restore these specific HFD-associated markers to healthy levels. Regarding target protection and modification, the vancomycin resistance gene *vanT* was reduced by 6.56% and tetracycline determinants including *tet(T)* (3.6% reduction), *tetB(P)* (4.3% reduction), and the regulator *TxR* (5.3% reduction) were suppressed; however, the novobiocin resistance marker novA showed a significant upward trend (184.6 to 197.6). Shifts in drug inactivation and regulation were similarly complex: the beta-lactamase gene *TaeA* decreased by approximately 5.2%, yet the transcriptional repressor mecI increased by 7.7% (119.2 to 128.4), and the neomycin resistance regulator NmcR remained significantly higher than the ND baseline. Consequently, while beLP1^®^ suppressed specific markers typically associated with HFD-induced stress, the expansion of these other subtypes resulted in a slight increase in the overall abundance of the Multidrug and Vancomycin resistance classes. These findings indicate a targeted remodeling of the genetic reservoir rather than a restoration of resistome balance or a consistent overall reduction in the resistome reservoir.

## 4. Discussion

### 4.1. Postbiotic Modulation of Gut Metabolic Function

The beLP1^®^ intervention did not completely normalize body weight gain or fasting blood glucose levels to the extent reported for some live probiotic strains. Several probiotic strains, including *L. plantarum* HMRS-6 and *Bifidobacterium longum* APC1472, have been reported to attenuate body-weight gain in HFD-fed mouse models [25,26]. Unlike live probiotics, which may actively colonize the gut and compete for dietary calorie absorption or directly metabolize sugars, heat-killed preparations primarily interact with host receptors via structural components to modulate inflammation [11]. Consequently, beLP1^®^ appears to exert its primary metabolic benefits by mitigating hepatic lipid overload [17], reducing specific visceral fat depots, and reducing low-grade inflammation, rather than driving massive systemic weight loss. Multiple *Lactobacillus* species such as *L. plantarum* [25], *L. paracasei* [27], *L. rhamnosus* [27], and *L. fermentum* [28] have demonstrated anti-obesity benefits, including reductions in body weight, improvements in glucose and insulin responses, and modulation of lipid and hepatic metabolism. Similar effects have also been observed with heat-killed *Lactobacillus* and *Bifidobacterium* strains [29]. This reduction suggests that beLP1^®^ may play a role in regulating lipid metabolism under obesogenic conditions. Earlier reports showed that *B. longum* subsp. *infantis* FB3-14 supplementation significantly reduces overall body and adipose mass [30]. These divergent outcomes underscore the highly strain-specific and state-specific (heat-killed vs. live cells) nature of postbiotic interventions [31]. The convergence of body weights by Week 10 may be due to the continuous high-caloric pressure of the 60% kcal HFD, which can eventually overwhelm the transient suppression of weight gain observed in the early phase. The significant reduction in ALT, AST, and triglycerides despite comparable final body weights suggests that beLP1^®^ promotes a ‘metabolically healthy’ profile by reducing ectopic lipid accumulation and protecting liver function.

The functional metagenomic analysis demonstrates that the administration of beLP1^®^ selectively promotes a metabolic shift characterized by a significantly enhanced capacity for Acetate production. This finding is critical, providing strong evidence that the shifts in bacterial abundance are directly correlated with an enhanced genetic capacity for SCFA synthesis (Figure 4). Although beLP1^®^ supplementation resulted in a modest reduction in alpha diversity compared to the ND group, this outcome reflects the targeted enrichment of specific beneficial taxa rather than a detrimental loss of commensals. The massive expansion of keystone SCFA-producing species, particularly *A. muciniphila* and *Faecalibaculum sp.*, mathematically reduces community evenness [32]. When a few keystone species become highly dominant, community evenness inherently drops [33]. In this context, the lower alpha diversity signifies a therapeutic restructuring of the microbiome, where a few highly functional species become dominant to establish a protective metabolic environment against HFD-induced dysbiosis. A notable decrease in *Streptococcus* species was observed in the beLP1^®^ group, suggesting a reduction in taxa associated with inflammation and metabolic dysfunction [34]. *C. cocleatum* has been linked to dysbiotic states and inflammatory processes in metabolic disorders [35,36], which was reduced in this study.

The dramatic enrichment of the acetate pathway potential in the HFD + beLP1^®^ group (accounting for approximately 30% of the predicted SCFA synthesis pathways, as depicted in Figure 5) is strongly supported by the concurrent, targeted proliferation of key acetate-producing taxa observed in the taxonomic analysis (Figure 4). The most striking finding is the substantial increase in *A. muciniphila*, which showed the highest positive shift among all measured species, exhibiting a log_2_ fold change of +1.3 (Figure 4). This represents an approximately a 2.5-fold enrichment over the HFD group.

As a keystone mucin-degrading bacterium, *A. muciniphila* actively ferments mucin to produce essential SCFAs, primarily acetate and propionate [37,38,39]. Therefore, the proportional increase in the predicted Acetate pathway potential directly reflects the enhanced metabolic activity and successful colonization of this keystone species under beLP1^®^ supplementation. Further evidence supporting this acetate and propionate dominant functional profile comes from the enrichment of other acetate and propionate -producing genera. *Faecalibaculum* sp. (log_2_ FC > +0.6) and *Lactococcus* spp. (log_2_ FC > +0.7) were significantly enriched in the HFD+ beLP1^®^ group (Figure 4). *Faecalibaculum rodentium*, an anaerobic member of the Erysipelotrichaceae family, is specifically recognized for its role in acetate production and maintaining gastrointestinal homeostasis [40]. Similarly, *Lactococcus* spp. are primary fermenters that produce acetate and lactate [41]. The concurrent expansion of these primary fermenters provides a robust taxonomic foundation for the maximal Acetate synthesis capacity observed in the functional analysis. While the significant expansion of *A. muciniphila* is typically associated with improved metabolic health, it is important to acknowledge its complex role in gut barrier homeostasis. Recent studies have indicated that in certain contexts, such as post-antibiotic recovery or low-fiber environments, over-colonization of *A. muciniphila* may lead to excessive mucin consumption and subsequent thinning of the mucus layer [42,43]. Although our study observed significant hepatoprotection via reduced ALT and AST, which often correlates with improved barrier function, the absence of direct histological or tight-junction protein analysis means that the impact of beLP1^®^ on the physical intestinal barrier remains to be confirmed.

This specific pattern of change indicates that the postbiotic mechanism of heat-killed beLP1^®^ is characterized not by reversing the gut function back to the ND profile, but by optimizing specific functional traits within the existing HFD-induced environment. The intervention leverages available mucin or residual carbohydrate substrates to maximize the output of primary fermentation products by selectively promoting specialized commensals, such as *A. muciniphila* and *Faecalibaculum*. The resultant functional enhancement of the acetate pathway is thus the direct, predictable metabolic consequence of beLP1^®^’s highly selective taxonomic remodeling, affirming the postbiotic’s role as a targeted agent.

### 4.2. Integrated Functional and Anti-Inflammatory Outcomes

The functional remodeling instigated by beLP1^®^ contributes to a broader anti-dysbiotic phenotype that aligns with the metagenome-predicted functional potential for SCFA biosynthesis. The substantial increase in *A. muciniphila* (a key mucin specialist) paired with the Acetate boost acts synergistically on gut barrier function. *A. muciniphila* directly enhances mucin synthesis by goblet cells [44], while acetate serves as an essential energy source for the epithelial layer, thus maintaining barrier integrity and reducing inflammation caused by the translocation of microbial products [45]. The functional genomic assessment showed that beLP1^®^ intervention led to the robust enrichment of major propionate precursor suppliers (*A. muciniphila*, *Faecalibaculum sp.*, and *Lactococcus* spp.). Although the beLP1^®^ intervention resulted in a lower relative proportion of predicted butyrate synthesis pathways, this trade-off appears to be compensated by the substantial, maximal boost in acetate and propionate potential. Acetate serves as a vital energy substrate for the epithelium and a precursor for microbial cross-feeding networks [46], while propionate strongly influences hepatic lipid metabolism and energy homeostasis. Furthermore, the concurrent enrichment of these beneficial bacteria contributes to a effectual lactate pool, another essential propionate precursor, thereby sustaining propionate synthesis flux via the acrylate pathway [47]. Given the concurrent suppression of pro-inflammatory taxa and the systemic improvements in metabolic markers, the relative reduction in butyrate does not appear to compromise overall gut health [48]. Instead, beLP1^®^ seems to establish an alternative, protective SCFA equilibrium tailored to the HFD-conditioned environment. This pattern indicates a functional remodeling centered predominantly on maximizing acetate generation capability alongside a high capacity for propionate precursor availability within the HFD-conditioned environment, which is highly relevant given propionate’s role in satiety and antilipogenic effects [48,49].

This shift towards a healthier metabolic and physical environment is further reinforced by the concomitant suppression of pro-inflammatory and dysbiotic taxa [50]. Notably, the administration of beLP1^®^ resulted in a substantial decrease in the relative abundance of *Clostridium cocleatum* (approximately −1-fold reduction) (Figure 4). *C. cocleatum* has similarly been reported to participate in dysbiosis-associated inflammation and metabolic imbalance. Similarly, the reduction in *Streptococcus* (−2 fold) and *Sporofaciens musculi* (−1.7 fold) alongside the decrease in the pathobiont *Mucispirillum schaedleri* all contribute to a gut environment characterized by reduced low-grade inflammation typical of HFD-induced obesity [51] (Figure 4).

### 4.3. Taxonomic Association of ARGs

Taxonomic binning linked the predominant ARGs to members of Enterobacteriaceae, Bacteroidaceae, and Lachnospiraceae, in agreement with prior resistome studies [52,53,54]. It is important to note that the close coupling between *Enterobacteriaceae* and ARG abundance is expected, partially due to the inherent bias in current ARG databases toward well-characterized taxa [54]. However, our shotgun metagenomic analysis adds valuable resolution by demonstrating how beLP1^®^ with HFD could selectively suppresses this specific resistance reservoir [55]. Within these families, *Escherichia coli* and *Bacteroides uniformis* were major carriers of multidrug efflux and glycopeptide resistance genes [56,57]. In contrast, HFD+ beLP1^®^-treated mice exhibited increased proportions of beneficial taxa such as *Faecalibaculum rodentium* and *L. murinus*, both of which have the potential to enhance gut barrier function or induce inflammation [58,59].

### 4.4. beLP1^®^ Restores Resistome Balance

Across studies, abundance changes in specific taxa closely track ARG shifts [55]. For example, in Shen et al., increases in genera like *Alistipes* or *Parabacteroides* went hand-in-hand with ARG fluctuations [55]. Our data mirror this: bacterial taxa that rose or fell with beLP1^®^ treatment carried many of the affected resistance genes. This indicates a microbiota-mediated mechanism of ARG regulation. Moreover, functional profiling suggested that pathways for antibiotic resistance were selectively dampened by beLP1^®^. The enrichment of efflux-associated genes is particularly important as they are often co-located with other resistance determinants and contribute to multidrug resistance phenotypes [60,61]. In particular, genes encoding antibiotic efflux pumps and two-component regulatory systems were downregulated in the probiotic group. These systems are well-known drivers of multidrug resistance [62], so their suppression implies that beLP1^®^ not only shifts community composition but also reprograms resistance functions. Thus, beLP1^®^ appears to exert both taxonomic and functional rebalancing, reducing ARG expression by reshaping the metabolic potential of the gut microbiome.

Although a highly diverse gut microbiome is typically a hallmark of good health, the slight drop in alpha diversity observed in the beLP1^®^ group likely reflects a targeted ecological shift rather than a harmful loss of commensal bacteria. Because keystone taxa such as *A. muciniphila* expanded so dramatically, this massive bloom mathematically lowered the overall ‘evenness’ of the community. Rather than a sign of dysbiosis, this points to the establishment of a specialized microbial environment better equipped to counter HFD-induced metabolic stress. This same restructuring naturally explains the mixed changes we observed in ARG abundance. beLP1^®^ does not simply eradicate the gut resistome; instead, it selectively suppresses high-risk pathogenic carriers, like certain *Enterobacteriaceae*. At the same time, it promotes beneficial commensals that inherently carry their own baseline resistance genes. Consequently, the relatively minor fluctuations in ARGs point to a specific, functional remodeling of the microbiome rather than a broad-spectrum clearing effect.

Overall, our findings demonstrate that dietary fat loading profoundly expands the gut ARG reservoir, but a non-viable probiotic intervention can partially reverse this effect. Rather than a uniform reduction, the beLP1^®^-mediated effect on the resistome is characterized by a complex pattern of gene-specific increases and decreases. This suggests that beLP1^®^ does not simply ‘shrink’ the resistome but shifts its composition, potentially by favoring the expansion of specific commensal populations that harbor different resistance profiles. Importantly, using a heat-killed preparation offers safety and stability advantages: the structural components of *L. plantarum* can modulate the microbiota without the risk of live bacterial translocation or gene transfer [22]. These results highlight heat-killed probiotics as a promising, low-risk strategy to mitigate diet-associated antibiotic resistance, and they suggest that targeting the microbiota is a viable approach to control the gut resistome.

The SCFA biosynthesis profiles reported in this study represent the metagenome-derived functional potential for acetate, propionate, and butyrate production. While these metagenomic predictions offer valuable mechanistic insights, the actual in vivo metabolite fluxes remain to be confirmed, as absolute SCFA concentrations in fecal, tissue, or plasma samples were not directly measured. In particular, directly quantifying butyrate would help clarify its specific contribution to the observed health benefits. Therefore, future studies incorporating targeted metabolomics (e.g., GC-MS) are warranted to evaluate absolute SCFA concentrations and validate these predictive findings.

## 5. Conclusions

In conclusion, heat-killed *L. plantarum* beLP1^®^ offers targeted metabolic benefits by mitigating HFD-induced hepatotoxicity and dyslipidemia, even though improvements in final body weight and fasting glucose were not statistically significant. Metagenomic profiling indicates that beLP1^®^ shifts the microbiome toward a diverse, anti-inflammatory state specifically enhancing the potential for acetate and propionate synthesis while inducing a selective modulation of specific ARG subtypes rather than a consistent overall resistome reduction. These findings support beLP1^®^ as a safe and effective postbiotic candidate for managing diet-induced metabolic stress, though future studies incorporating direct metabolite quantification are warranted to confirm these mechanistic pathways in vivo.

## Figures and Tables

**Table 1:** Representative ARG subtypes and their relative abundance in each group after 10 weeks of beLP1 administration.

No	ARG	ARG Type	Resistance Mechanism	ND	HFD	HFD + beLP1	Phylum
**1**	otr(A)	Tetracycline	Antibiotic efflux (MFS transporter)	99.2	88.8	88.4	Actinobacteria
**2**	arlR	Multidrug	Regulatory system (efflux control)	319	311.6	323.6	Firmicutes
**3**	arlS	Multidrug	Sensor kinase (regulation of efflux)	195.8	189.8	196.4	Firmicutes
**4**	arnA	Polymyxin	Lipid A modification (target alteration)	125.2	112.6	119.6	Proteobacteria
**5**	basS	Polymyxin	Two-component regulation system	295.8	355.6	366.2	Proteobacteria
**6**	bcrA	Multidrug	Antibiotic efflux (ABC transporter)	745.4	866.4	873	Firmicutes
**7**	cdeA	Multidrug	Antibiotic efflux (RND transporter)	368.8	432	426.4	Proteobacteria
**8**	efrA	Multidrug	Antibiotic efflux (ABC transporter)	162.4	185.6	188.8	Firmicutes
**9**	evgA	Multidrug	Regulatory system (efflux activation)	82.2	88.6	86.6	Proteobacteria
**10**	evgS	Multidrug	Sensor kinase (efflux regulation)	574	600.4	583.2	Proteobacteria
**11**	golS	Heavy metal	Regulatory system (efflux and resistance)	84	127	126.6	Proteobacteria
**12**	IreK	Multidrug	Regulatory system (kinase-mediated)	135.8	143.8	153	Firmicutes
**13**	macB	Macrolide	Antibiotic efflux (ABC transporter)	1255	1309.2	1326.8	Proteobacteria
**14**	MdtK	Multidrug	Antibiotic efflux (MATE transporter)	90.2	71.2	86.6	Proteobacteria
**15**	mecI	Beta-lactam	Transcriptional repressor of resistance genes	123.6	119.2	128.4	Firmicutes
**16**	mepA	Multidrug	Antibiotic efflux (MFS transporter)	182	205	211.8	Firmicutes
**17**	msbA	Multidrug	ABC transporter protein	314.4	355.4	349.6	Proteobacteria
**18**	mtrA	Multidrug	Transcriptional activator (efflux regulation)	164.8	166	175.4	Proteobacteria
**19**	mupA	Unknown	Unknown	183.6	178	188.6	Unknown
**20**	mupB	Unknown	Unknown	174.4	158.2	160	Unknown
**21**	murA	Fosfomycin	Target alteration (cell wall biosynthesis enzyme)	119	127	128	Firmicutes
**22**	NmcR	Beta-lactam	Beta-lactamase regulator	88.8	115.6	111.8	Proteobacteria
**23**	novA	Aminocoumarin	Target protection/alteration	163.8	184.6	197.6	Actinobacteria
**24**	oleC	Macrolide-like	Antibiotic efflux (ABC transporter)	132.4	147.8	173	Actinobacteria
**25**	optrA	Oxazolidinone	Ribosomal protection	140	131.6	129	Firmicutes
**26**	patB	Fluoroquinolone	Antibiotic efflux (MFS transporter)	119	124.2	121.8	Firmicutes
**27**	PmrF	Polymyxin	Target alteration (lipid A modification)	325.2	256.6	282.8	Proteobacteria
**28**	ramA	Multidrug	Global regulator of efflux systems	106.2	114.8	130	Proteobacteria
**29**	RanA	Aminoglycoside	ABC-type efflux pump	297.2	385	381	Proteobacteria
**30**	rpoB2	Rifampin	Target alteration (RNA polymerase beta-subunit)	153.4	144.4	144	Actinobacteria
**31**	smeS	Multidrug	Antibiotic efflux (RND system)	69	98.6	114.4	Proteobacteria
**32**	soxS	Unknown	Unknown	150.2	159.4	184.8	Unknown
**33**	TaeA	Beta-lactam	Beta-lactamase (enzyme degradation)	134	92.6	87.8	Proteobacteria
**34**	tet(T)	Tetracycline	Ribosomal protection protein	166.6	163.4	157.6	Firmicutes
**35**	tetA(58)	Tetracycline	Antibiotic efflux (MFS transporter)	335.6	407.8	412.6	Proteobacteria
**36**	tetB(P)	Tetracycline	Antibiotic efflux (MFS transporter)	100.6	92	88	Firmicutes
**37**	TxR	Tetracycline	Regulatory system (efflux control)	167	151.2	143.2	Firmicutes
**38**	vanR	Vancomycin	Regulatory system (van operon)	1000.8	1124.2	1226.4	Firmicutes
**39**	vanS	Unknown	Unknown	349.2	376.4	407.4	Unknown
**40**	vanT	Vancomycin	Cell wall modification	109	125.2	117.6	Firmicutes

Values are expressed as absolute normalized abundance using TPM (Transcripts Per Million), representing quantitative gene load rather than relative percentages (0–100%).

## Data Availability

The original contributions presented in this study are included in the article/Supplementary Material. Further inquiries can be directed to the corresponding authors.

## Supplementary Materials

The following supporting information can be downloaded at: https://www.mdpi.com/article/10.3390/microorganisms14050944/s1, Figure S1: Animal experimental design and treatment groups; Figure S2: Effect of beLP1 on body weight in HFD-induced obese mice; Figure S3: PERMANOVA analysis between three different groups. Figure S4: Comparison of relative taxa abundance distribution (species level) between HFD and HFD + beLP1 groups after 10 weeks of beLP1 administration. Bar graph shows significant changes in beneficial (A) and pathogenic (B) taxa. Figure S5: eggNOG COG functional composition across groups and time points.
