# Supplementary material for: Shotgun Metagenomic Analysis of Gut Microbiota and Antibiotic Resistance Genes in a High-Fat Diet Mouse Model Treated with Heat-Killed Lactiplantibacillus plantarum beLP1
Source: Microorganisms. 2026 Apr 22;14(5):944. doi: 10.3390/microorganisms14050944 (PMC13209379; doi:10.3390/microorganisms14050944)
Supplement: Supplementary file 1 [file microorganisms-14-00944-s001.zip › microorganisms-4228898-supplementary.pdf]

Shotgun Metagenomic Analysis of Gut Microbiota and Antibiotic Resistance Genes in a High-Fat Diet Mouse Model Treated with Heat-Killed *Lactiplantibacillus plantarum* beLP1

Ranjith Kumar Manoharan[1,*,†], Hyun-Dong Shin[1,†], Yura Lee[1], Sunhwa Baek[1], Eunjung Moon[1], Youn Bum Park[1], Junhui Cho[1], Im-Joung La[2], Dong Ha Lee[3], Kwon-Il Han[1], Sathiyaraj Srinivasan [4,*]

[1]Research & Development Center, Bereum Co., Ltd., Wonju 26361, Republic of Korea

[2]Atomy R&D Center, Gongju 32511, Republic of Korea

[3]Dx&Vx Co., Ltd., Geumchoen-gu, Seoul 08513, Republic of Korea

[4]Department of Bio & Environmental Technology, College of Natural Science, Seoul Women's University, 623 Hwarangno, Nowon-gu, Seoul 01797, Republic of Korea

*Corresponding author: mrkumar@bereum.com (R.K.M.); drsrini@swu.ac.kr (S.S.)

†These authors contributed equally to this work.

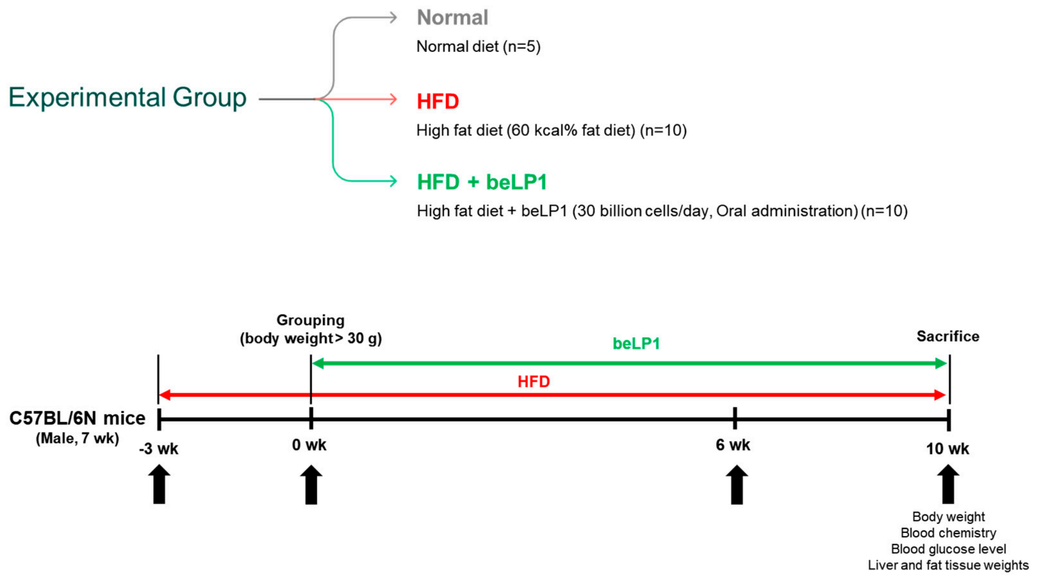

Supplementary Figures

Figure S1. Animal experimental design and treatment groups

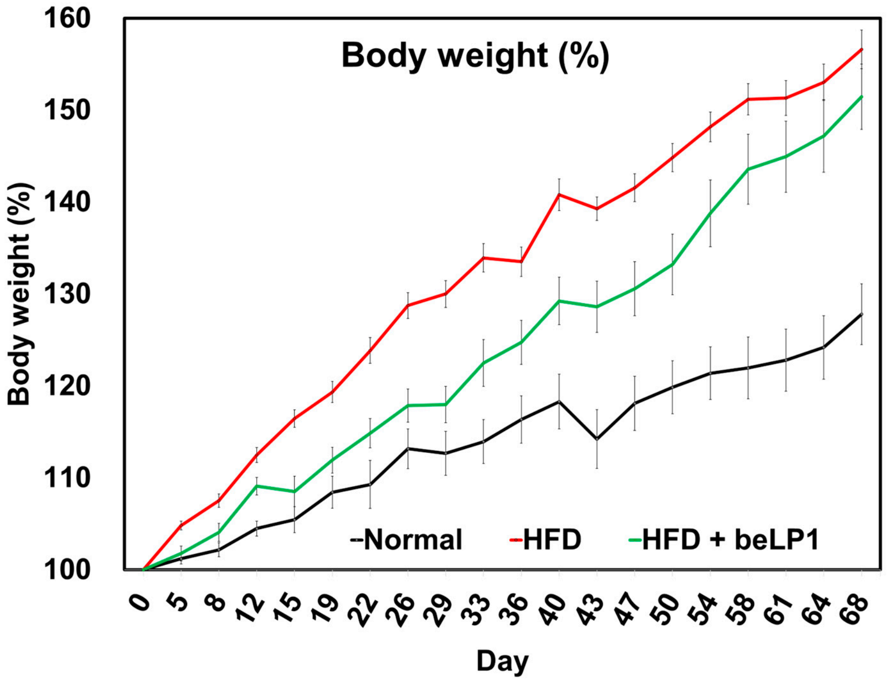

Figure S2. Effect of beLP1 on body weight in HFD-induced obese mice

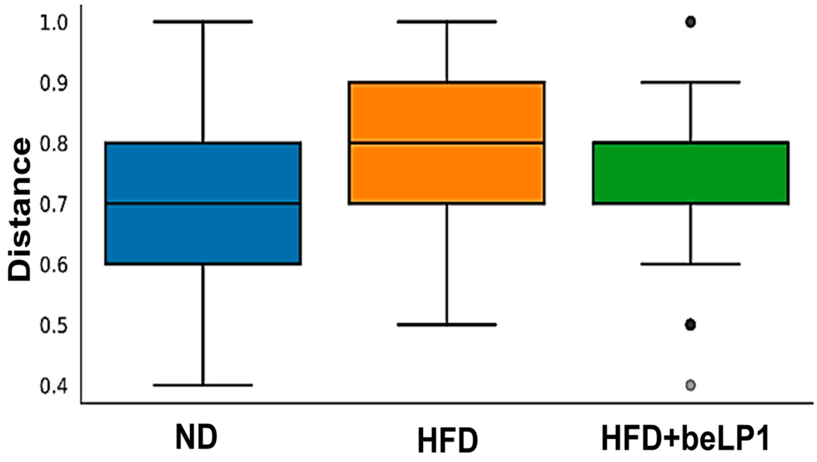

Figure S3. PERMANOVA analysis between three different groups

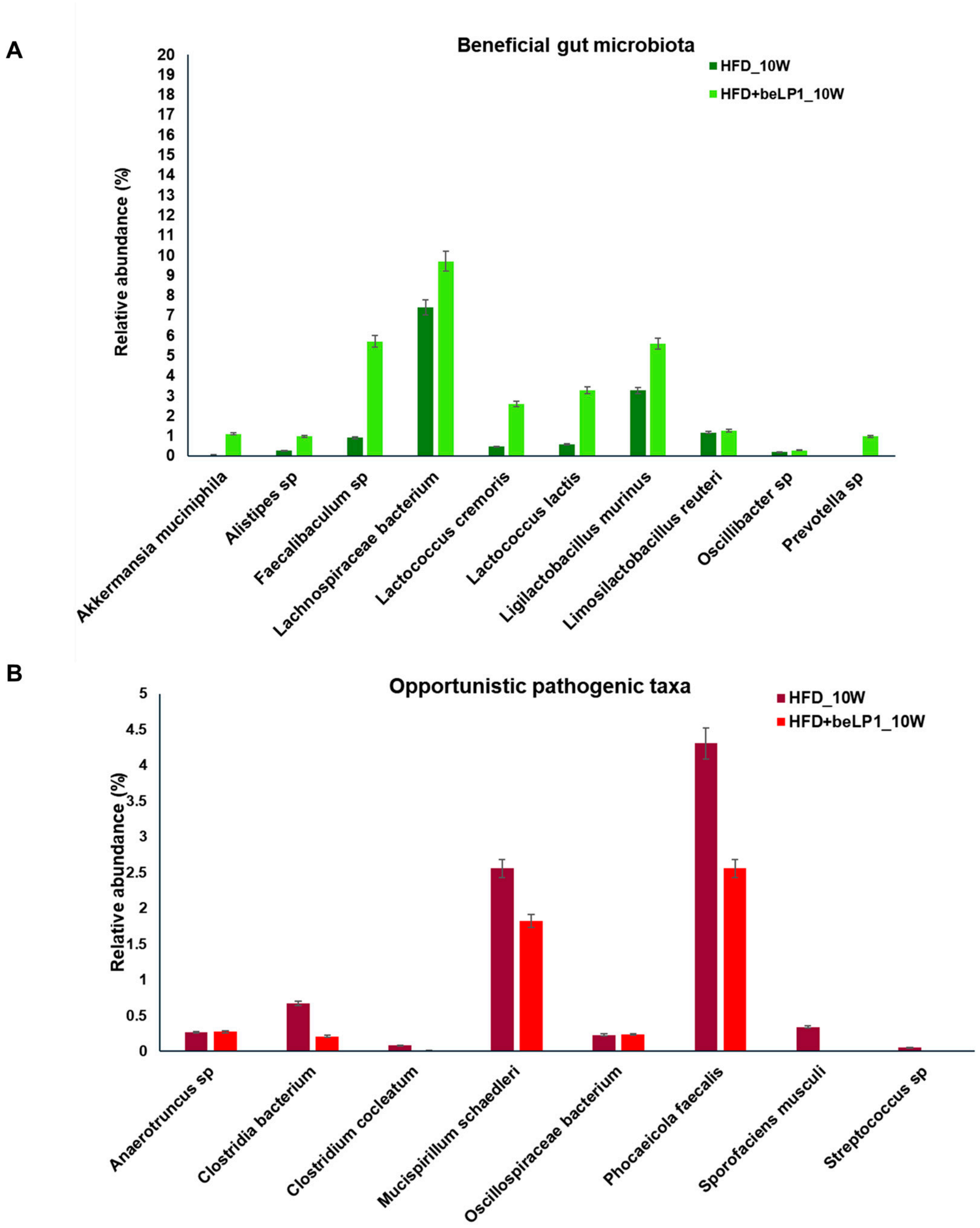

Figure S4. Comparison of relative taxa abundance distribution (species level) between HFD and HFD+beLP1 groups after 10 weeks of beLP1 administration. Bar graph shows significant changes in beneficial (A) and pathogenic (B) taxa.

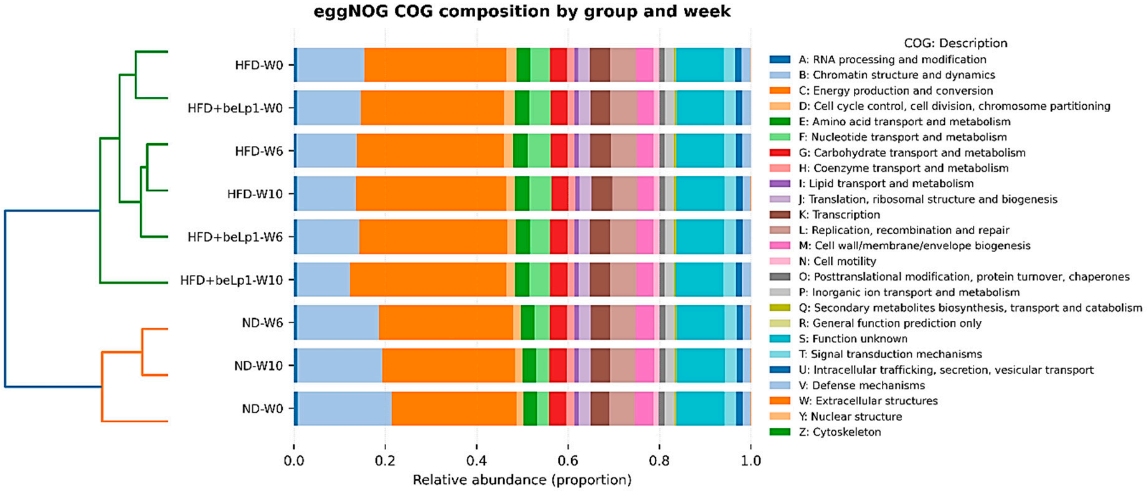

Figure S5. eggNOG COG functional composition across groups and time points. The stacked bar chart illustrates the relative abundance of genes categorized into 23 Clusters of Orthologous Groups (COG) functional categories across all experimental groups (ND, HFD, and HFD +beLP1) at baseline (W0), Week 6 (W6), and Week 10 (W10)
